# Correction: Simulating the Real Origins of Communication

**DOI:** 10.1371/journal.pone.0117638

**Published:** 2015-03-06

**Authors:** The PLOS ONE Staff

## Notice of Republication

This article was republished on December 15, 2014, to fix incorrect characters in the General Methods section and missing italicization throughout the article. The publisher apologizes for the errors. Please download this article again to view the correct version. The originally published, uncorrected article and the republished, corrected article are provided here for reference.

## Supporting Information

S1 File Originally published, uncorrected article. (PDF)

S2 File Republished, corrected article. (PDF)
