# Supplementary material for: Simulating the Real Origins of Communication
Source: PLoS One. 2014 Nov 26;9(11):e113636. doi: 10.1371/journal.pone.0113636 (PMC4245210; doi:10.1371/journal.pone.0113636)
Supplement: Document S1 — Model definition. (PDF) [file pone.0113636.s001.pdf]

Document S1. Model definition

R. A. Blythe and T. C. Scott-Phillips
Supplement to "Simulating the real origins of communication"

The simulation model is defined on a two-dimensional square lattice of $L \times L$ sites with periodic boundary conditions. At the beginning of each generation, F distinct sites are chosen at random and populated with food and N agents are randomly assigned a location in the system (more than one agent may initially reside on the same site). Food remains stationary until the next generation; agents can move according to the rules set out below. In the first generation all agents are assigned common values of four parameters referred to as motility $m \in [0, 1]$, directionality $d \in [-1, 1]$, brightness $b \in [0, 1]$ and contrast $c \in [-1, 1]$, and are taken to belong to the same species. Each agent is equipped with a light which initially is not activated.

In each elementary update, an agent is selected at random. With probability m this agent moves to a neighbouring site: for this purpose all eight sites that lie adjacent to an agent in the horizontal, vertical or diagonal directions are considered neighbours. If movement is to occur, and at least one agent of the same species is showing a light, then with probability $|d|$ the selected agent moves onto the neighbouring site that is closest to (if $d > 0$) or furthest away from (if $d < 0$) the nearest light emanating from an agent of the same species. Otherwise, the movement direction is random. Whether or not movement occurs, the agent's light is switched off and the target site is inspected. If this site contains food, one unit of food is consumed by the agent, and with probability $\frac{b}{2}(1 + c)$, the light is switched on. Otherwise if the site does not contain food, the light is switched on with probability $\frac{b}{2}(1 - c)$. N iterations of this elementary update defines a unit of time known as a Monte Carlo sweep. The generation ends after T such sweeps.

At the end of a generation, each agent i is assigned a fitness

$$s_i = f_i \exp\left[-\kappa(b_i + |c_i| + m_i + |d_i|)\right] \tag{S1.1}$$

where f_i is the number of food units consumed by the agent, and κ is a positive parameter that ascribes a fitness cost to larger (absolute) values of the parameters b_i,c_i,m_i and d_i that together define the strategy of agent i. N offspring agents are then created by repeatedly sampling (with replacement) and copying a parent agent from the population in such a way that agent i is chosen to be a parent with a probability proportional to s_i. When the copy is made, there is a probability μ that one of the parameters b_i, c_i, m_i, d_i is replaced with a value sampled uniformly from the appropriate range (the remaining parameters are copied from a parent). In 'free' simulation conditions, any of these four parameter values can be chosen for replacement. Meanwhile, in 'bounded' simulation conditions, one of three parameter values can be chosen for replacement: the excluded parameter is c_i in the case where a reaction is pre-specified, and d_i in the case where an action in pre-specified. Each mutant individual created in this way is taken to be the founder member of a new species (i.e., its lights will be visible only to non-mutant offspring of this newly-created individual). These N offspring agents then form the population for the next generation of the simulation.

In the 'natural' simulation condition, the value of f_i is set to zero for each agent at the start of each generation. In the 'neutral' simulation condition, the score for each agent is set to a value drawn from the distribution

$$p(f) = \begin{cases} 1 - \lambda(b) & \text{if } f = 0 \\ \frac{L^2\lambda(b)^2}{FT} \exp\left(-\frac{L^2\lambda(b)f}{FT}\right) & \text{for } f > 0 \end{cases} . \tag{S1.2}$$

The form of the function $\lambda(b)$ was determined empirically by setting up simulations in which agents can only move around the lattice randomly, i.e., with $b = c = d = 0$ and some $m \in [0, 1]$. The distribution of the food consumed was obtained numerically, and found to fit the above distribution for some choice of λ that depended on m. The functional dependence of $\lambda(m)$ was found to fit $\lambda(m) = 1 - \alpha e^{-\beta m}$ for some combination of α and β (see below for the values that applied in our simulations). By replacing the dependence on m with a corresponding dependence on b, we ascribed to brightness the same intrinsic fitness benefit of moving around the lattice randomly to consume food, as described in the main text.

The simulation ends after G generations. The final state of the population is characterised by the action strength $A = b^* c^*$ and reaction strength $R = m^* d^*$ where the starred parameters correspond to the strategy of the most abundant species in the population.
