# Supplementary material for: Simulating the Real Origins of Communication
Source: PLoS One. 2014 Nov 26;9(11):e113636. doi: 10.1371/journal.pone.0113636 (PMC4245210; doi:10.1371/journal.pone.0113636)
Supplement: Document S2 — Simulation parameters and conditions. (PDF) [file pone.0113636.s002.pdf]

Document S2. Simulation parameters and conditions

In all simulations, we took $L = 51$, $N = 200$, $F = 10$, $\kappa = 0.25$, $\mu = 0.01$, $T = 1000$ and $G = 1000$. The simulation conditions differed in the initial values of b, c, d and m that characterised the entire population, whether one of the parameters could not be changed by mutation, and whether the intrinsic fitness advantage of moving around the lattice was neutralised. More precisely in Experiment 1, the agents always had their food consumption f reset to zero at the start of each generation, and varied only in the following dimensions:

- *No communication* — initial $b = c = d = m = 0$; one of the four parameters is changed in each mutation event.
- *Pre-specified reaction, free condition* — initial $b = 0$, $c = m = d = 1$; one of the four parameters is changed in each mutation event.
- *Pre-specified reaction, bounded condition* — initial $b = 0$, $c = m = d = 1$; one of the three parameters b, m or d is changed in each mutation event (c is never changed).
- *Pre-specified action, free condition* — initial $m = 0$, $b = c = d = 1$; one of the four parameters is changed in each mutation event.
- *Pre-specified reaction, free condition* — initial $b = 0$, $c = m = d = 1$; any parameter could be changed in mutation.
- *Pre-specified action, bounded condition* — initial $m = 0$, $b = c = d = 1$; one of the three parameters b, c or m is changed in each mutation event (d is never changed).

In Experiment 2, the natural conditions were as described above. For each of these natural conditions, there is a corresponding neutral condition which differs only in that each agent has its food consumption f initialised to a value drawn from (S1.2) at the start of each generation. For the combination of L, F and T given above, we found that the empirical parameters $\alpha = 0.968$ and $\beta = 1.91$ that appear in the function $\lambda(m)$ specified in the previous section gave a good fit to the distribution of f obtained in the mobility-only benchmark simulations.
