# Supplementary material for: Simulating the Real Origins of Communication
Source: PLoS One. 2014 Nov 26;9(11):e113636. doi: 10.1371/journal.pone.0113636 (PMC4245210; doi:10.1371/journal.pone.0113636)
Supplement: Document S3 — Mathematical model. (PDF) [file pone.0113636.s003.pdf]

Document S3. Mathematical model

We present a refinement of the mathematical model presented in [1] that allows the differences and similarities in actions and reactions to be understood in more precise terms.

As in [1], we use t, a and r to represent specific states of the world, actions and reactions respectively. The world is assumed to spend a fraction $\phi(t)$ of its time in state t, $p(a|t)$ is the probability an agent will respond to state t by performing action a, and $q(r|a)$ is the probability an agent will react to action a with reaction r. When the reaction is performed, both actor and reaction may receive a payoff that depends on the reaction performed and the state of the world. The actor receives a payoff $\Pi_A(r|t)$ and the reactor a payoff $\Pi_R(r|t)$ that may differ from the actor's. As discussed in more detail in [2], we also assume that there is an intrinsic cost $\epsilon(a)$ for maintaining the ability to perform the action a, and an intrinsic cost $\epsilon(r)$ for maintaining the ability to perform the reaction r. As in both [1] and [2], we take $\epsilon(a_0) = \epsilon(r_0) = 0$, where a_0 and r_0 correspond to some default (non-communicative) action and reaction respectively.

One way to characterise the system is in terms of the stability of specific actions and reactions. This is established by identifying the fitness $u(a,t)$ associated with the strategy of responding to state t with action a, and the fitness $v(r,a)$ associated with the strategy of reacting to action a with reaction r. Then, when $u(a',t) > u(a,t)$ for some pair of actions a and a', a mutant species that uses action a' in response to t will outcompete a wild-type species that uses action a (assuming all other action and reaction strategies are the same).

For the reaction, we use the same set-up as in [1], and take

$$v(r,a) = \sum_t \phi(t)\Pi_R(r|t)p(a|t) - \epsilon(r) \ . \tag{S3.1}$$

For actions we introduce a small modification, in that we allow in addition to any communicative benefit of performing an action, an intrinsic benefit $\alpha(a|t)$. Then, the fitness of the action strategy is

$$u(a,t) = \phi(t)\left[\alpha(a|t) + \sum_r \Pi_A(r|t)q(r|a)\right] - \epsilon(a) \ . \tag{S3.2}$$

The intrinsic benefit $\alpha(a|t)$ was referred to verbally in [1], but not explicitly included in the mathematical treatment in that work. We find its explicit inclusion is valuable for a number of reasons. Most importantly, it proves a formal protocol for establishing whether a state of communication, as defined in [1], exists. This is achieved by choosing $\alpha(a|t)$ in such a way that an action or reaction becomes pre-specified without communication being present. Then, we let the system evolve to a steady state, at which point we set all $\alpha(a|t) = 0$. If an action-reaction pair is then maintained in the absence of the original impetus for the action or reaction, we say that both the resulting system and that with nonzero $\alpha(a|t)$ are communicative. This protocol is similar to what is done in the simulation model, where the initial condition is set up so as to have an action or reaction already in place. Moreover, we can use it to demonstrate that there are stronger constraints on realising an initial condition where a reaction is pre-specified rather than an action, as was argued in [1]. At the same time, we find that once an action or reaction is pre-specified, there is no further fundamental asymmetry between them, and as such it should be possible to neutralise any difference between them that is found in a specific system (as was achieved in the simulation). Finally, the inclusion of the $\alpha(a|t)$ parameters allows us to identify when some component of an agent's behaviour (movement, in the simulation model) should be formally regarded as an action, rather than a reaction.

A. Pre-specifying an action or a reaction

To pre-specify an action in this framework, we need there to be some action $a \neq a_0$ that has an intrinsic benefit in some state of the world $t \neq t_0$. That is

$$\alpha(a|t) > \frac{\epsilon(a)}{\phi(t)} \quad \text{for some } a \neq a_0 \text{ and } t \neq t_0 \tag{S3.3}$$

as our initial condition. As a consequence, $u(a,t) > u(a_0,t)$, and so strategy $t \to a$ becomes established: thus, an initial action is pre-specified. For communication to evolve, two further conditions must be satisfied. First, some reaction $a \to r$ where $r \neq r_0$ must emerge. This occurs if $v(r,a) > v(r_0,a)$, i.e., if

$$\phi(t)\left[\Pi_R(r|t) - \Pi_R(r_0|t)\right] > \epsilon(r) \quad \text{for some } r \neq r_0 \ . \tag{S3.4}$$

Finally, for this to be a *bona fide* communicative interaction, it must persist once the original reason for the action is removed, which is achieved by setting $\alpha(a|t) = 0$. The action is maintained by the presence of the reaction if we still have $u(a|t) > u(a_0,t)$, i.e., if

$$\phi(t)\left[\Pi_A(r|t) - \Pi_A(r_0|t)\right] > \epsilon(a) \ . \tag{S3.5}$$

The condition (S3.4) ensures that the reaction is maintained due to the continued presence of the action. Thus the action and reaction are mutually sustaining, as required for communication [1].

To pre-specify a reaction instead is more complicated. Since, by definition, a reaction can provide a payoff when it is a response to an action, this means that some action must also be present in the initial condition. In turn, this action must in itself have some intrinsic benefit. Moreover, we will find below that the state of the world in which this action is initially performed must be distinct from that in which the action forms part of a truly communicative interaction. The need for such an additional *incidental* state of the world is ultimately responsible for pre-existing reactions to be *a priori* less likely than pre-existing actions in the origins of real communication systems.

The mathematical conditions for communication via a reaction-first scenario are as follows. First, we require an action, a, which exists in some incidental state of the world t'. This will arise if

$$\alpha(a|t') > \frac{\epsilon(a)}{\phi(t')} \quad \text{for some } a \neq a_0 \text{ and } t' \neq t_0 \tag{S3.6}$$

as above. In order now to pre-specify a non-communicative reaction $a \to r$, we must have

$$\phi(t')\left[\Pi_R(r|t') - \Pi_R(r_0|t')\right] > \epsilon(r) \quad \text{for some } r \neq r_0 \ . \tag{S3.7}$$

For communication to emerge, we require the action a to be performed in some state of the world $t \neq t_0$ (but need not necessarily differ from t_i). This happens if

$$\phi(t)\left[\Pi_A(r|t) - \Pi_A(r_0|t)\right] > \epsilon(a) \quad \text{for some } t \neq t_0 \ . \tag{S3.8}$$

For true communication, we further require that reaction is retained once its initial impetus is removed by setting $\alpha(a|t') = 0$. This leads to the further condition

$$\phi(t)\left[\Pi_R(r|t) - \Pi_R(r|t)\right] > \epsilon(r) \ . \tag{S3.9}$$

As before, the action and reaction become mutually reinforcing when these conditions hold.

We now compare the conditions under which communication is established by pre-specifying the action, (S3.3)–(S3.5), with those that arise from pre-specifying the reaction, (S3.6)–(S3.9). We see immediately that all three conditions for an action-first scenario also apply to the reaction-first scenario, but the latter has the extra condition (S3.7). An exception to this would be if the incidental state of the world t' coincides with the state t in which communication is stable. However, in this case, the action $t \to a$ necessarily emerges before the reaction, and hence we require $t' \neq t$ to realise the reaction-first scenario. Hence, the conditions under which a reaction can be pre-specified are necessarily more restrictive than those under which an action can be pre-specified.

B. Failure to establish communicative strategies

The foregoing discussion concerned *necessary* conditions for communication to emerge. These may not, however, be sufficient. For example, once an action is initially established, it is not enough for a communicative reaction to grow in the presence of this action: it must also grow more rapidly than any other reaction. Expressed mathematically, we must not have a reaction r' such that

$$\phi(t)\left[\Pi_R(r'|t) - \Pi_R(r'|t)\right] > \epsilon(r') - \epsilon(r) \ . \tag{S3.10}$$

If there is such a reaction, and we also have

$$\phi(t)\left[\Pi_A(r'|t) - \Pi_A(r_0|t)\right] < \epsilon(a) \ , \tag{S3.11}$$

the action $t \to a$ will not survive once the initial impetus for the action is removed, i.e., if $\alpha(a|t)$ is set to 0, and indeed may not do so even if this impetus is maintained. In this situation, a reaction that is beneficial to the reactor but detrimental to the actor has emerged, which in turn causes the action strategy to be eliminated.

A corresponding 'exploitation' effect is also possible in the reaction-first scenario. Here, we need a state $t'' \neq t, t'$ where the benefit to the actor of performing the action a exceeds that of performing the same action in t. For this to be true, we need

$$\phi(t'')\Pi_A(r|t'') > \phi(t)\Pi_A(r|t) \ . \tag{S3.12}$$

To prevent a communicative state from being reached, the reaction r must be detrimental to the reactor in the environment t'' (relative to doing nothing):

$$\phi(t_e)\left[\Pi_R(r|t'') - \Pi_R(r_0|t'')\right] < \epsilon(r) \ . \tag{S3.13}$$

This then eliminates the pre-existing reaction.

The point of this analysis is to show that the conditions (S3.10) and (S3.11) are quite different from (S3.12) and (S3.13). Therefore, there is no reason to expect that the case where an action is pre-specified is typically more or less prone to a communicative strategy failing to evolve than the case where a reaction is pre-specified.

In Experiment 1 in the main text, we found that restricting the ability to use lights unreliably had a significant effect on the probability of establishing communication when the reaction was pre-specified, but restricting the ability to move in a random direction had little effect when the action was pre-specified. This result can be understood from the foregoing analysis. If lights are not used reliably, there is a considerable disadvantage to the reactors: here there is a state of the world t'' which comprises an agent showing its light despite not being on food. This carries little or no cost for the actor (i.e., both sides of (S3.12) are equal or roughly equal), in which case the reaction strategy can drift in. If it does so, the cost for reactors moving to lights where this is no

food is much worse than diffusing around the lattice, and so the pre-existing reaction is rapidly eliminated.

There is no such corresponding effect in the action-first scenario: there is no way for an agent to move so as to cause considerable disadvantage to an actor who is reliably showing their light. Hence, in this particular system, a pre-existing action is more likely to evolve to a state of full communication than a pre-existing reaction. However, systems where the converse is true are mathematically possible.

C. Classification of behaviour into action and reaction

In the initial set-up of the simulation model, it was natural to interpret the lighting behaviour as corresponding to an action, and the movement behaviour as a reaction. However, it transpires that only part of the movement behaviour—systematic motion towards (or away from) the nearest light—counts as a reaction. The random component of the movement is in fact an action, as it can contribute to an agent's fitness in the absence of communication. The only way to account for this within the mathematical framework above is to assign a nonzero $\alpha(a|t)$ for an action a that corresponds to random motion at a given rate.

This assignment furthermore makes clear the inherent asymmetry between movement and lighting that was noted in the main text: there is no such term for any component of the lighting behaviour in its own right (i.e., without communication taking place). The manipulation introduced in the neutral condition amounts to adding a similar term for an action that corresponds to switching the light on whether or not food is present. Once this is done, the movement and lighting behaviours are on a more equal footing, and it is then the case that the propensity to evolve communicative behaviour with either as a starting point is much more similar.

---

[1] Scott-Phillips TC, Blythe RA, Gardner A, West SA (2012) How do communication systems emerge? Proc Roy Soc B 279: 1735.

[2] Scott-Phillips TC, Blythe RA. (2013) Why is combinatorial communication rare in the natural world, and why is language an exception to this trend? J Roy Soc Interface 10: 20130520.
