# Supplementary material for: Correction: Simulating the Real Origins of Communication
Source: PLoS One. 2015 Mar 6;10(3):e0117638. doi: 10.1371/journal.pone.0117638 (PMC4352057; doi:10.1371/journal.pone.0117638)
Supplement: S1 File — (PDF) [file pone.0117638.s001.pdf]

PLOS ONE

RESEARCH ARTICLE

Simulating the Real Origins of Communication

Richard A. Blythe[1]**, Thomas C. Scott-Phillips**[2*]

1. School of Physics and Astronomy, University of Edinburgh, Edinburgh, United Kingdom, **2.** Department of Anthropology, Durham University, Durham, United Kingdom

*t.c.scott-phillips@durham.ac.uk

These authors contributed equally to this work.

Abstract

How communication systems emerge is a topic of relevance to several academic disciplines. Numerous existing models, both mathematical and computational, study this emergence. However, with few exceptions, these models all build some form of communication into their initial specification. Consequently, what these models study is how communication systems transition from one form to another, and not how communication itself emerges in the first place. Here we present a new computational model of the emergence of communication which, unlike previous models, does not pre-specify the existence of communication. We conduct two experiments using this model, in order to derive general statements about how communication systems emerge. The two main routes to communication that we identify correspond with findings from the empirical literature on the evolution of animal signals. We use this finding to explain when and why we should expect communication to emerge in nature. We also compare our model to experimental research on the origins of human communication systems, and hence show that humans are an important exception to the general trends we observe. We argue that this is because humans, and probably only humans, are able to 'signal signalhood', i.e. to express communicative intentions.

CrossMark
click for updates

OPEN ACCESS

Citation: Blythe RA, Scott-Phillips TC (2014) Simulating the Real Origins of Communication. PLoS ONE 9(11): e113636. doi:10.1371/journal.pone.0113636

Editor: Stephanie Ann White, UCLA, United States of America

Received: June 10, 2014

Accepted: October 28, 2014

Published: November 26, 2014

Copyright: © 2014 Blythe, Scott-Phillips. This is an open-access article distributed under the terms of the Creative Commons Attribution License, which permits unrestricted use, distribution, and reproduction in any medium, provided the original author and source are credited.

Data Availability: The authors confirm that all data underlying the findings are fully available without restriction. Raw data are supplied as Supplementary Information.

Funding: This work was supported by Research Councils UK (RB) and Economic and Social Research Council (TSP). The funders had no role in study design, data collection and analysis, decision to publish, or preparation of the manuscript.

Competing Interests: The authors have declared that no competing interests exist.

Introduction

Communication is a critical source of information for organisms, which allows them to successfully navigate their local ecology, including their social environment. Correspondingly, the origins and evolution of communication is a central topic for several disciplines (e.g. behavioural ecology, artificial life, cognitive science), and has implications for many others, including the human

behavioural sciences [1–7]. Many existing models study and seek to explain various aspects of this emergence [2, 3, 5, 8]. One particularly fertile area of study is the use of computational and mathematical models to study the origins and evolution of human language (see e.g. [9–11] for reviews).

However, with few exceptions (see below), these models all build in the fact of communication from the outset, and in doing so preclude study of how communication emerges from scratch. Specifically, previous models typically pre-define at least one of the following: the communication channel; the roles of signaller and receiver; or the forms that signals and/or responses can take. For example, in one representative study, some agents were labelled as 'transmitters', who sent either a '0' or '1' signal; and other agents, labelled 'receivers', then used the signal to determine between two distinct behaviours [12]. In short, both the roles of signaller and receiver were pre-defined, as were the forms that signals and responses can take. Consequently, this model does not (and is not able to) study the emergence of communication, and the conditions that give rise to it. Instead, it investigates whether and how an already existing system will evolve to take a different (more optimal) form. The same is true of the vast majority of existing mathematical and computational models.

In order to simulate the actual origins of communication itself, we should, in contrast to this previous work, define only a set of possible actions for the agents, and investigate the conditions under which these actions take on communicative functions (i.e. become either signals or responses). There is one existing computational simulation that does this [13]. Here, simulated robots evolved a rudimentary form of communication, despite the fact that none of the above aspects of communication were pre-defined. However, this research did not explore the conditions under which communication would and would not emerge; it simply showed that it is in principle possible to study this emergence. As such, this research "showed the potential for… models to look at the real origin of communication" ([14], italics added) – but this potential has not been exploited. In particular, neither that study, nor any other subsequent research, investigated the specific conditions under which communication would and would not emerge from an initial non-communicative state. In short, no study to date has experimentally investigated the "real" origins of communication (Figure 1).

Here we present a new computational simulation designed to address these issues. Specifically, we present a stochastic, computational simulation in which agents would benefit from communication, but where communication itself is not pre-specified. Instead, the agents simply have behaviours they can perform, or not. In other words, we provide only the most basic 'equipment' for communication to occur, but do not pre-specify whether or how this equipment should be used. In this way, we investigate the "real" origins of communication, in the sense that no communicative function is built into any behaviour a priori, and must instead evolve (just as was the case in ref. [13]). The key question we seek to address is whether there are any general statements that can be made about how communication systems emerge. To answer this question, we run the simulation

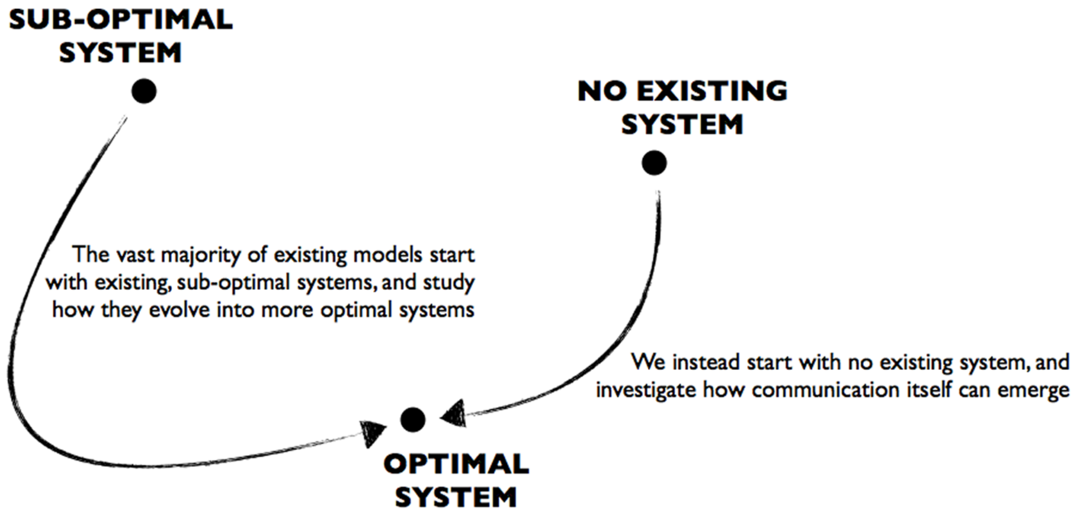

Figure 1. Comparison with previous research. The vast majority of research on the evolution of communication to date has studied how pre-existing systems evolve to take on other, typically more optimal forms. Here, we instead investigate how behaviour can evolve to take on a communicative function in the first place.

doi:10.1371/journal.pone.0113636.g001

in a number of different experimental conditions. This allows us to investigate in a controlled way the conditions under which behaviours take on the roles of signals and responses; in other words, the conditions under which communication does and does not emerge. Otherwise, the simulation is designed to be as simple as possible, in order to make interpretation of the results tractable. In the discussion we link our results to the empirical literatures on (i) the evolution of animal signals, and (ii) experimental research on the origins of human communication systems.

General Methods

Here, we describe the general set-up of the simulations. Full details are provided in Document S1.

Mobile agents inhabit a two-dimensional square lattice, which contains (inexhaustible) sources of food at random locations. Each agent has a light, which can be turned on or off. If the agent is located on a food source, it consumes food, effectively adding to its fitness. Agents move around the lattice in a way determined by a strategy that may vary from one individual to another (see below). Each generation lasts for a fixed number of timesteps, after which agents reproduce asexually, with mutation, in a way that the expected number of offspring an individual had is proportional to the amount of food consumed. Mutation involves the replacement, with probability μ, of one of the parameters b, c, m, or d (see below), with a different value drawn at random from the corresponding distribution.

A particular agent's strategy is determined by the values of four parameters, as follows:

- Movement is determined by the two parameters $m \in [0,1]$ ('mobility') and $d \in [-1,1]$ ('directionality'). An agent moves to one of its eight neighbouring lattice sites with probability m. If an agent does move, its direction must be determined. With probability $|d|$ this direction is towards (if $d>0$) or away from (if $d<0$) the nearest light source emanating from an agent of the same species; otherwise it moves in a random direction. In short, the mobility parameter determines the overall propensity to move, and the direction parameter determines the propensity for movement to be a specific direction.
- In a similar way, the use of lights is determined by the two parameters $b \in [0,1]$ ('brightness') and $c \in [-1,1]$ ('contrast'). An agent is programmed to use its light with probability b. If an agent does use its light, exactly what environmental circumstances it will turn the light on for must be determined. With probability $|c|$ the outcome of this decision is determined by the presence (if $c>0$) or absence (if $c<0$) of food; otherwise the light is turned on at random. In short, the brightness parameter determines the overall propensity for the light to be used, and the contrast parameter determines the propensity for the state of the light to be informative about the presence or absence of food.

In this way, lights can potentially be used as signals, to indicate the presence of food, but, crucially, this is not something that is necessarily pre-defined. Instead, whether the lights are used at all, whether that use correlates with the presence of food, and whether other agents attend to the lights, depends on the values of the four parameters described above. To measure this, we define the variable A (action) as the product of b and c, and the variable R (reaction) as the product of m and d. If both are equal or close to 1, we can say that communication exists. By using two rather than one parameter for each behaviour, we separate an overall propensity to perform the behaviour from the part of the behaviour that plays a role in communication. This will allow us to directly observe the emergence of these propensities.

Each agent's light is visible only to other agents of the same species i.e. only those with the same parameter values. This means that, although dishonesty (the use of lights when not on food) and information suppression (the non-use of lights when on food) are possible, there is no incentive to either once communication is established, because a mutant individual who does one of these things will not obtain any benefit from the majority behaviour in the population at large.

The inclusion of a mechanism of this sort is a reasonable one given our goals, and given the ubiquity of communication in the natural world, where communication must be more-or-less stable much of the time (it is of course possible that some natural occurring cases of communication are unstable, but it is vanishingly unlikely that this is true in general). How stability is ensured is a much studied question for the evolution of communication [2, 4]. However,

questions of stability are orthogonal to questions of emergence, and so we neutralise issues of stability, in order to directly study the conditions under which initially non-communicative behaviours will evolve into signals and responses. We choose kin discrimination to do this only because it is easy to implement, and allows us to observe the emergence of communication straightforwardly. (Note also that notions of dishonesty and information suppression only make sense if a communication system actually exists; for this reason, we use the term 'unreliability' as a more general term to describe the use of lights when not on food, whether or not a communication system actually exists.) We discuss the effects of this manipulation in the Discussion.

It is important to be clear that, while there is only one viable mode of communication in this design, that does not mean that the fact of communication is itself pre-specified. The task we are interested in is not how to choose between multiple different modes of communication. Instead, we wish to ask whether and how communication will emerge, given the existence of behaviours that could in principle be used to communicate. As such, the inclusion of only one viable mode of communication is the right approach, since it removes other factors that might complicate our analysis. In this way, our design is formally analogous to the Embodied Communication Game, a task used to experimentally investigate how pairs of interacting humans create new systems of communication [15]. There too, there was only one viable mode of communication, but whether and how human participants were actually able to make use of it was a non-trivial question. After we have presented the results of our simulation, we will compare them to the results of this previous experimental work.

Experiment 1: Two processes of emergence

To establish a baseline for subsequent manipulations, we first ran the simulation with initial parameter values $b=c=m=d=0$ (and so $A=R=0$ also). As with all manipulations described below, we performed 500 runs of the simulation, each for 1,000 generations. In this baseline condition communication never emerged, even though it would have been adaptive: the most abundant species in the final state has values of b, c, m and d that were all close or equal to 0, and at no point did any of these values exceed 0.1. This result is consistent with the findings of our previous mathematical model, which showed that a state of no interaction is evolutionarily stable [16].

Next, we ran the simulation with initial parameter values that effectively pre-specified the existence of either actions or reactions. Specifically, we first ran the simulation with initial parameter values that pre-specify that agents move towards lights: $c=m=d=1$, $b=0$ i.e. a pre-specified reaction. We did this under two different experimental conditions. In the 'free' condition, mutations cause any one of the four parameters (b, c, m, d) to change (each with equal probability). The 'bounded' condition is identical, except that the parameter $c=1$ is kept fixed throughout. This ensures that lights only ever signal food i.e. that lights cannot ever be used unreliably. The purpose of this comparison between free and

bounded conditions is to provide a measure of the extent to which, despite the fact that there is no selective pressure to use lights unreliably (see above), the possibility that lights might be used unreliably (e.g. because of drift) affects the probability that communication will emerge. To do this, we take the most abundant species at the end of each simulation, calculate A for this species, and then take two measurements of A: its mean final value over the different runs within each condition; and its full distribution over the different different runs within each condition. These measurements can then be compared across different conditions.

We also ran the simulation with with initial parameter values that pre-specify that agents turn on lights when on food: $b=c=d=1$, $m=0$ i.e. a pre-specified action. Again, we did this under a free condition (all parameters liable to mutation) and a bounded condition ($d=1$ is kept fixed throughout). In this case, the bounded condition ensures that, if agents do travel around the lattice, they will only ever travel towards lights, rather than at random. Here, the comparison between free and bounded conditions provides a measure of the extent to which the possibility that agents might ignore lights affects the probability that communication will emerge. We can measure this is in the same ways as described above, but for R rather than A.

In sum, experiment 1 used a 2×2 design: bounded vs. unbounded crossed with pre-specified actions vs. pre-specified reactions. The two main questions we wished to ask were: (i) Does the possibility that the behaviours in question (movement; turning on lights) might be used 'unreliably' affect the probability that communication will emerge, independently of any effects they have on communication itself?; and (ii) Is communication more likely to emerge when actions or reactions are pre-specified, or are these two sets of initial conditions equivalent in this respect? Question (i) will be answered by the free vs. bounded comparison, and question (ii) will be answered by the pre-specified actions vs. pre-specified reactions comparison. Since the sample size is quite large (500 independent simulation runs for each condition), it is appropriate to use a two-sided two-sample z-test to compare the mean final values of A and R. (Although the underlying distribution of A and R are not normal, the central limit theorem implies that the distributions of their means are, and hence tests assuming such normality can be used.).

Results are summarised in Figure 2. As can be seen, the difference between the mean final values of A (conditions in which the reaction is pre-specified) is highly significant ($p=4.71\times10^{-19}$), whilst the difference between the mean final values of R (conditions in which the reaction is pre-specified) is not ($p=0.175$). To compare the full distributions of these final values of A and R, we used a Kolmogorov-Smirnov (K-S) test, which makes no assumptions about the form of the distribution. We find similar results i.e. a highly-significant difference when the reaction is pre-specified ($p=1.78\times10^{-30}$), and no difference when the action is pre-specified ($p=0.172$). This means that the answer to question (i), above, is yes, but only for pre-existing reactions. In other words, the possibility that lights might be used unreliably diminishes the likelihood that communication will

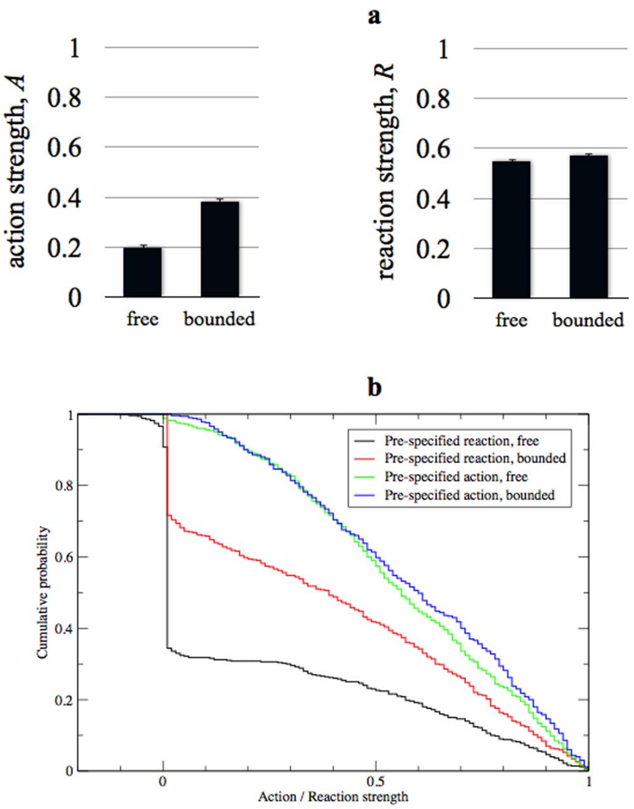

Figure 2. Results of Experiment 1. Figure 2a shows the mean action and reaction strengths at the end of the simulations in Experiment 1, under both free and bounded conditions ('free'='all parameters subject to mutation'; 'bounded'='one parameter fixed to ensure that unreliability is impossible'). Figure 2b shows the cumulative distributions of action and reaction strengths, under the same conditions, such that when one line lies above another, the quantity under investigation is skewed to higher values. Both figures show that the bounded manipulation makes no difference to the likelihood that communication will emerge when the action pre-exists, but it does make a difference when the reaction pre-exists.

doi:10.1371/journal.pone.0113636.g002

emerge, even when agents have no incentive to use lights unreliably – but there is no similar result for 'unreliably' following lights. Question (ii) should also be answered in the affirmative: communication is more likely to emerge when actions are pre-specified, rather than reactions. Moreover, this difference is not fully explained by the results above: there is a highly significant difference between the pre-specified actions and pre-specified reactions (z-test: $p=4.55\times10-23$; K-S test: $p=3.20\times10-23$; these results are for the bounded condition only).

Taken together, this pair of results suggests that, although the relative potential of prospective 'signals' and 'responses' to be used unreliably is one factor that affects the emergence of communication, it is not the only factor. We thus designed a second experiment to investigate what other factors are at play.

Experiment 2: Emergence depends on adaptive value of prior behaviour

We hypothesised that the reason why communication was more likely to emerge when actions rather than reactions were pre-specified was that equal propensities to manipulate the light (brightness, b) and to move around the system (mobility, m) may not lead to equal payoffs when communication is absent. To illustrate, compare a hypothetical agent X, which has $m \neq 0$ (and so moves randomly in the absence of lights), with a hypothetical agent Y, which has $m=0$ (and so simply does not move at all). In the absence of lights, agent X will explore a greater proportion of the lattice than Y, and hence is more likely to find food by chance. Compare now the hypothetical agents W, which has $b \neq 0$ (and so turns on light when on food), and Z, which has $b=0$ (and so does not turn on their light). Here, in the absence of other agents moving towards lights, agent W does no better (and no worse) than agent Z. What this pair of comparisons show is that an increased propensity to move can be adaptive regardless of any effect this might have on the emergence of communication – but there is no equivalent result for turning on lights. Effects of this general sort are implicit in some other models. If this verbal analysis is correct, there is a greater incentive to move than to turn on the light when communication is not established, and this could in turn increase the probability that communication will emerge if actions are pre-specified, relative to the probability that communication will emerge if reactions are pre-specified. A manipulation that neutralises this difference should equalise the corresponding probabilities.

To test this, we ran four further conditions. In experiment 1, agents' scores were set to zero at the start of each generation (call these 'natural' conditions). We instead ran 'neutral' conditions, in which agents' scores are set at the start of each generation to a value drawn from distribution that describes the scores that agents will accrue by moving around the lattice without interacting with other agents (we obtained this distribution by sampling the amount of food consumed by agents for given values of m, and ascribing scores from the distribution so obtained to agents with the corresponding value of b; see Document S2 for details). We did this for the same four conditions as in experiment 1 (i.e. both free and bounded, for both pre-specified actions and pre-specified reactions).

The results supported our analysis (Figure 3). Under the neutral manipulation, the means of all four distributions are more similar to each other, and closer to the values previously found when the action was pre-specified. Most importantly, in contrast to experiment 1, there is no significant difference between the case of a pre-specified action and that of a pre-specified reaction under either the free (z-test: $p=0.479$; K-S test: $p=0.902$) or bounded condition (z-test: $p=0.0771$; K-S test: $p=0.226$) when the neutral manipulation was imposed. This confirms that the reason for the difference between pre-specified actions and pre-specified reactions in Experiment 1 is the adaptive value of increased movement in the absence of communication, as proposed above. What this result illustrates is that whether the pre-existing reaction is adaptive itself, or is adaptively neutral, will

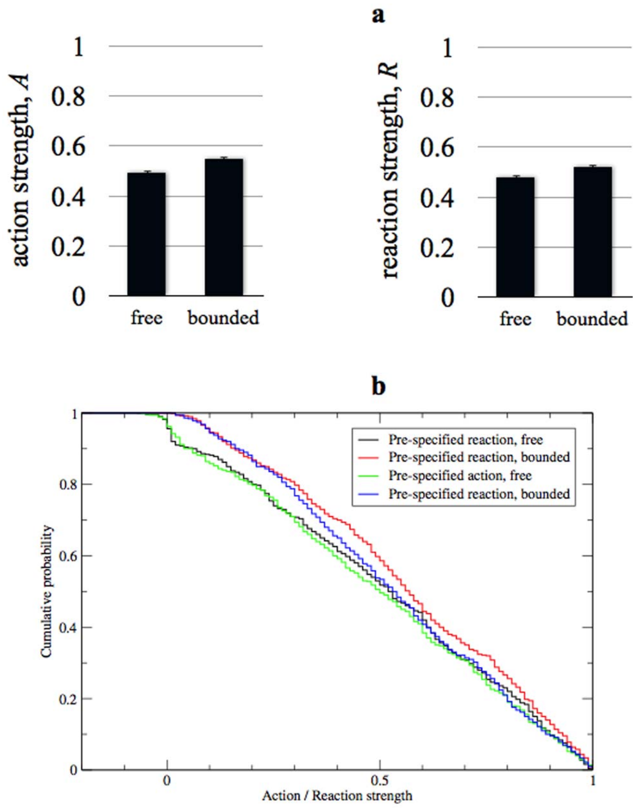

Figure 3. Results of Experiment 2. As with Figure 2, this figure shows both the mean action and reaction strengths at the end of the simulations (Figure 3a), and the culmulative distributions of action and reaction strengths (Figure 3b). Here, unlike Figure 2, there is no marked difference between equivalent conditions with pre-specified actions and pre-specified reactions. As we highlight in the Discussion, these results, when combined with the results of Experiment 1 (see Figure 2), can explain why there are more reports in the empirical literature of the emergence of communication in some ways rather than others.

doi:10.1371/journal.pone.0113636.g003

affect the probability than communication will emerge. We see no reason why this same point could not in principle apply to pre-existing actions too. In light of this, we present, in Document S3, a refinement of our previous mathematical model [16], to take this factor into account.

General Discussion

Taken as a whole, our results suggest a number of general statements that can be made about how communication systems emerge. First, there are two processes by which communication can emerge by natural selection: pre-existing actions or pre-existing reactions (without either of these, communication did not emerge). Second, although these two process are mathematically equivalent to one another in most respects, they differ in (at least) one important way, namely that pre-specification of reactions is more liable to collapse due to issues of unreliability than is pre-specification of actions. Third, the relative probability of each process

is also affected by the adaptive value of the pre-existing actions or reactions are adaptive.

Our approach here was inspired by and built upon our previous mathematical model [16]. That model was based upon the abstract mathematical framework of evolutionary game theory, and made several simplifying assumptions (e.g. spatial mixing; no demographic stochasticity; all agents having equal access to the same environmental states, the same signals, and so on; and others). By using an agent-based model, we have been able in this paper to investigate the same topic experimentally, and in a more concrete system i.e. one in which each agent has its own autonomy, where there is stochasticity of various relevant sorts, and so on. In this way, our results endorse and expand upon the findings of our previous model. Specifically, our models here endorse the finding of our previous model that the emergence of communication requires pre-existing actions or reactions; and they expand upon our previous model by highlighting the fact that the probability that these pre-existing behaviours will actually become signals/responses depends upon their relative contribution to fitness. In this way, our models provide a way to disentangle how different aspects of behaviour contribute to different aspects of the process of emergence (the emergence of signals, the emergence of responses, and the contribution each makes to fitness). We have formalised these differences in the updated version of our mathematical model provided in Document S3.

Most models concerned with the evolution of communication focus on issues of stability. We abstracted away from these issues, in order to focus on the orthogonal topic of emergence (see Introduction). If dishonesty had been permitted in our model, would it have completed matters, and led to different results? Our previous mathematical model suggested that the potential for dishonesty would have one specific effect: it would make it more likely that communication would emerge when the action is pre-specified, rather than the reaction (i.e. by a process of ritualization, rather than sensory manipulation; see below) [16]. The reason for this is that in the former case the actor must already receive some benefit from the pre-existing action, independent of the effects the action has on the reactor (otherwise the action would not exist) – but in the latter case there is no equivalent foundation: there is no reason why the reactor must receive any prior benefit from the pre-existing reaction. This result also holds in our new mathematical model, which updates our previous model in light of the findings in this paper (see Document S3 for the updated mathematical model). Further research on the exact nature of the interaction between emergence and stability is warranted.

How do our results relate to the empirical literature on the evolution of animal signals? This literature has established two processes by which communication systems can emerge, and these two processes correspond to the two processes we have identified in our model. The first is ritualization, in which agents evolve to take advantage of the fact that some other agent's behaviour correlates with (i.e. is informative about) some aspect of the world. In other words, an action pre-exists, and this provides the selective environment for the evolution of a reaction. One

classic example is the use of urine to signal territorial boundaries, which originated from animals leaving their territory to urinate [2]. The other process is sensory manipulation, in which the opposite occurs: a reaction pre-exists, and this provides the environmental context for the evolution of a 'signal'. An example is the offering of nuptial gifts, from males to females, that occurs in many insect species; in many cases, the female has a pre-existing mechanism that prioritised the opportunity to feed on large prey, and so the presentation of food gave the male an opportunity to mate (ibid.). Our results describe the initial conditions necessary for these two processes to occur.

How do our results relate to experimental research on the origins of human communication systems? (For reviews of this literature, see refs. [6, 17].) Like the existing modelling literature on the emergence of communication, most experimental studies with humans pre-define important aspects of the communicative interaction itself [15]. However, there are a small number of studies that do not do this [15, 18, 19]. In these studies, participants are faced with a task that is formally equivalent to our simulation with initial parameter values $b=c=m=d=0$ (experiment 1). However, unlike this condition in our simulation, in which communication did not emerge, the participants in these experiments were able to create new communication systems. How can this difference be explained? The participants in this and similar experiments succeeded because they were able to communicate the fact that they were trying to communicate; in other words, they were able to 'signal signalhood'. This allowed them to create new systems without the prior existence of actions or reactions. This ability to signal signalhood requires sophisticated forms of social cognition, in particular rich metacognition i.e. on the ability to reason about others' intentions, beliefs, and so on [7, 19–21]. It is quite possible, even likely, that no other species has these abilities to the extent that is required here [22]. If true, this can explain the differences in the results of those studies and the simulations presented here [7].

We would to conclude by reiterating that the distinctive feature of our model in this paper was that the existence of communication is not pre-specified in any way (see Introduction). To our knowledge, only one previous model did this [13]. However, that model was not used to experimentally test the different conditions under which communication would emerge. We have done this, and have hence been able to study the conditions under which non-communicative behaviour might take on a communicative function: the real origins of communication.

Supporting Information

Document S1. Model definition.
doi:10.1371/journal.pone.0113636.s001 (PDF)

Document S2. Simulation parameters and conditions.
doi:10.1371/journal.pone.0113636.s002 (PDF)

Document S3. Mathematical model.
doi:10.1371/journal.pone.0113636.s003 (PDF)

Author Contributions

Conceived and designed the experiments: RB TSP. Performed the experiments: RB. Analyzed the data: RB. Wrote the paper: RB TSP.

References

1. **Hauser MD** (1996) The Evolution of Communication. Cambridge, MA: MIT Press.
2. **Maynard Smith J, Harper DGC** (2003) Animal Signals. Oxford: Oxford University Press.
3. **Oller DK, Griebel U, editors** (2004) Evolution of Communication Systems: A Comparative Approach. Cambridge, MA: MIT Press.
4. **Searcy WA, Nowicki S** (2005) The Evolution of Animal Communication: Reliability and Deception in Signaling Systems. Princeton, NJ: Princeton University Press.
5. **Nolfi S, Mirolli M** (2010) Evolution of Communication and Language in Embodied Agents. Berlin: Springer.
6. **Galantucci B, Garrod S** (2011) Experimental semiotics: A review. Front Hum Neurosci 5: 11.
7. **Scott-Phillips TC** (2014) Speaking Our Minds. London: Palgrave MacMillan.
8. **Skyrms B** (2010) Signals: Evolution, Learning, and Information. Oxford: Oxford University Press.
9. **Nowak MA, Komarova NL** (2001) Towards an evolutionary theory of language. Trends Cogn Sci 5(7): 288–295.
10. **Kirby S** (2002) Natural language from artificial life. Artif Life 8(2): 185–215.
11. **Steels L** (2011) Modeling the cultural evolution of language. Phys Life Rev 8(4): 339–356.
12. **Oliphant M** (1996) The dilemma of Saussurean communication. BioSystems 37(1): 31–38.
13. **Quinn M** (2001) Evolving communication without dedicated communication channels. In: Kelemen J, Sosík, P, editors, Advances in Artificial Life: ECAL6. Berlin: Springer. pp. 357–366.
14. **Arranz J, Noble J, Silverman E** (2011) The origins of communication revisited. In: Lenaerts T, et al., **editors., Advances in Artificial Life: ECAL11.Cambridge, MA: MIT Press. pp. 47–54.**
15. **Scott-Phillips TC, Kirby S, Ritchie GRS** (2009) Signalling signalhood and the emergence of communication. Cognition 113(2): 226–233.
16. **Scott-Phillips TC, Blythe RA, Gardner A, West SA** (2012) How do communication systems emerge? Proc R Soc Lond B 279: 1943–1949.
17. **Scott-Phillips TC, Kirby S** (2010) Language evolution in the laboratory. Trends Cogn Sci 14(9): 411–417.
18. **de Ruiter JP, Noordzij ML, Newman-Norland S, Newman-Norland R, Hagoort P, et al.** (2010) Exploring the cognitive infrastruture of communication. Int Stud 11(1): 51–77.
19. **Stolk A, Verhagen L, Schoffelen JM, Oostenveld R, Blokpoel M, et al.** (2013) Neural mechanisms of communicative innovation. PNAS, 110(36): 14574–14579.
20. **Sperber D, Wilson D** (1995) Relevance: Communication and Cognition (2nd ed.). Oxford: Blackwell.
21. **Sperber D** (2000) Metarepresentations in an evolutionary perspective. In: Sperber D, (editor,), Metarepresentations: An Interdisciplinary Perspective. Oxford: Oxford University Press. pp. 117–137.
22. **Scott-Phillips TC** (2014) Non-human primate communication, pragmatics, and the origins of language. Curr Anthro. In press.
